# Enhancing Nutritional Contents of *Lentinus sajor-caju* Using Residual Biogas Slurry Waste of Detoxified Mahua Cake Mixed with Wheat Straw

**DOI:** 10.3389/fmicb.2016.01529

**Published:** 2016-10-13

**Authors:** Aditi Gupta, Satyawati Sharma, Ashwani Kumar, Pravej Alam, Parvaiz Ahmad

**Affiliations:** ^1^Department of Chemistry, St. Stephen’s College, Delhi UniversityDelhi, India; ^2^Centre for Rural Development and Technology, Indian Institute of Technology DelhiNew Delhi, India; ^3^Metagenomics and Secretomics Research Laboratory, Department of Botany, Dr. Harisingh Gour University Central UniversitySagar, India; ^4^Biology Department, College of Science and Humanities, Prince Sattam Bin Abdulaziz UniversityAl Kharj, Saudi Arabia; ^5^Department of Botany and Microbiology, College of Science, King Saud UniversityRiyadh, Saudi Arabia; ^6^Department of Botany, Sri Pratap CollegeSrinagar, India

**Keywords:** biogas slurry, detoxified mahua cake, *Lentinus sajor-caju*, saponins, wheat straw

## Abstract

Residual biogas slurries (BGS) of detoxified mahua cake and cow dung were used as supplements to enhance the yield and nutritional quality of *Lentinus sajor-caju* on wheat straw (WS). Supplementation with 20% BGS gave a maximum yield of 1155 gkg^-1^ fruit bodies, furnishing an increase of 95.1% over WS control. Significant increase (*p* ≤ 0.05) in protein content (29.6-38.9%), sugars (29.1-32.3%) and minerals (N, P, K, Fe, Zn) was observed in the fruit bodies. Principle component analysis (PCA) was performed to see the pattern of correlation within a set of observed variables and how these different variables varied in different treatments. PC1 and PC2 represented 90% of total variation in the observed variables. Moisture (%), lignin (%), celluloses (%), and C/N ratio were closely correlated in comparison to Fe, N, and saponins. PCA of amino acids revealed that, PC1 and PC2 represented 74% of total variation in the data set. HPLC confirmed the absence of any saponin residues (characteristic toxins of mahua cake) in fruit bodies and mushroom spent. FTIR studies showed significant degradation of celluloses (22.2-32.4%), hemicelluloses (14.1-23.1%) and lignin (27.4-39.23%) in the spent, along with an increase in nutrition content. The study provided a simple, cost effective approach to improve the yield and nutritional quality of *L. sajor-caju* by resourceful utilization of BGS.

## Introduction

Over the past few years, biogas has become an attractive renewable energy source in many nations across the globe (Chandra et al., 2011; Gupta et al., 2012; Barik and Murugan, 2015). Besides cow dung (CD), the traditional raw material, different kinds of agricultural products, viz., leaf litter, seed cakes, grasses, straw, husk, aquatic plants, biomass residues, etc., alone or in combination with CD, have also been evaluated for their potential to produce biogas (Gupta et al., 2011; Chaikitkaew et al., 2015; Chanakya et al., 2015; Cotana et al., 2015). After the anaerobic digestion process is complete, a large amount of residual biogas slurry (BGS) is obtained, whose management and disposal is a major problem and needs to be judiciously addressed (Liu et al., 2009; Hamelin et al., 2011). BGS are often too dilute but contain relatively high percentage of readily available nutrients, growth hormones and enzymes (Hamelin et al., 2011). Because of their nutrient content, BGS based farming technologies are becoming an important aspect of biogas dissemination programs which can replace energy intensive chemical fertilizers, improve crop yield as well as soil quality and fertility on a sustainable basis.

BGS can be applied directly in liquid or dried forms, as basal and top plant dressings, to enhance crop yield and soil fertility (Garg et al., 2005; Feng et al., 2011; Stumpe et al., 2012). Besides improving the soil and crop characteristics, many authors have reported other valuable applications of BGS. Jothi et al. (2003) observed that 10% application of BGS from a CD based biogas plant significantly enhanced the number and yield of tomato plants along with decreasing the nematode population in soil. Liu et al. (2009) concluded that biogas manure from agricultural wastes can be a valuable source of nutrients for soilless production of vegetables along with efficient means to decrease the building up of harmful substances (nitrates, etc.) in them. Namasivayam and Yamuna (1995) reported the removal of chromium (VI) and various dyes from wastewater using BGS as the adsorbent. Few studies have also been carried out on the use of BGS for mushroom cultivation. Gangulli and Chanakya (1994) evaluated that addition of CD based BGS to paddy straw, in 1:1 ratio, significantly enhanced the yield of *Lentinus flabellatus*, as compared from 100% paddy straw. Banik and Nandi (2004) reported improved yield and nutritional content of *Volvariella volvacea* and *Pleurotus sajor-caju* mushrooms from rice straw supplemented with residual BGS of poultry litter, cattle dung, municipal solid wastes and jute caddis.

Mushroom cultivation offers a cost effective and an eco-friendly method for efficient disposal of agricultural wastes along with producing proteinaceous food (Bisaria et al., 1987; Gothwal et al., 2012; Gupta et al., 2013a; Marlina et al., 2015). Popularity of oyster mushrooms (*Lentinus* ssp.) is growing as they can be cultivated effortlessly and economically on a variety of lignocellulosic agro-wastes, with high yield and nutritional values (Bisaria et al., 1987; Gothwal et al., 2012; Marlina et al., 2015). Recently, it has been reported that biological efficiency (BE) of the traditional substrates can be further enhanced by supplementation with residual BGS manures, thereby increasing the profitability of mushroom cultivation (Gangulli and Chanakya, 1994; Banik and Nandi, 2000, 2004). Amirta et al. (2016) reported two step usage of Shorea wood waste biomass: *P. ostreatus* cultivation and subsequent biogas production from mushroom spent mixed with CW. This combination gave a new industrial application of wood waste from tropical rain forests of Indonesia, along with producing value-added foodstuff and renewable energy.

In the present study, wheat straw (WS) was supplemented with residual BGS of detoxified mahua seed cake (DMC) and CD to enhance the yield and nutritional quality of *L. sajor-caju* mushrooms. Mahua seed cake (MC) is an important by-product of *Madhuca indica* tree, generated after the expulsion of oil from its seeds. It is rich in sugars, proteins but contains toxic saponins (Gupta et al., 2012). We have already reported biogas production from raw and DMC (Gupta et al., 2013a). Detoxification by simple water treatments gave cold water treated DMC (CW) and hot water treated DMC (HW), with significantly improved biogas yields over raw MC. BGS from three best combinations, i.e., 50 (HW DMC):50 (CD), 75 (HW DMC):25 (CD), 50 (CW DMC):50 (CD) were selected for the study and comparisons were made with CD slurry and WS control. To the best of our ability, this is the first study that reports the cultivation of *L. sajor-caju* on WS supplemented with residual BGS of DMC.

## Materials and Methods

### Substrates Procurement and Preparation

*Lentinus sajor-caju* (LS 1610) [old name *Pleurotus sajor-caju*] grain spawn was procured from Bharat Mushroom Organization, New Delhi, India. WS was chopped into 2-3 cm pieces and soaked overnight in water containing formalin, to ensure sufficient moisturization and complete sterilization. Subsequently, it was dried under shade to obtain an average moisture content of 60 ± 1%. CD slurry (T_1_) was procured from a running biogas plant at Micro-model, IIT Delhi, India. BGS of three combinations, viz., 50 (CW DMC):50 (CD) (T_2_), 50 (HW DMC):50 (CD) (T_3_), and 75 (HW DMC):25 (CD) (T_4_) were obtained from 5 L batch biogas reactors containing DMC and CD. The solid residues of these BGS were dried in shade and grounded. Saponin content in BGS- T_2,_ T_3_, and T4 was determined by extracting respective biogas slurries with 80% methanol followed by partitioning into the butanol phase (Gupta et al., 2013a,b). The dried BGS were powdered (maximum size 5 mm) and autoclaved at 121°C and 15 psi for 20 min, prior to their use.

### Experimental Design

Wheat straw (wet weight 2.5 kg, equivalent to 1 kg dry straw; moisture content 60%) was uniformly supplemented with 10, 20, and 30% BGS (T_1_, T_2_, T_3_, and T_4_), on dry weight basis. BGS were used as a source of nitrogen. Perforated polythene bags (28 cm × 20 cm) were used to pack these substrate combinations after subsequent inoculation with 10% *L. sajor-caju* grain spawn. The control composed of 100% WS with 10% grain spawn and 8% gram powder (added as a nitrogen source). All treatments were replicated in sets of three. Culturing conditions were maintained as mentioned in (Gupta et al., 2013b).

Harvesting of fruit bodies was done when the inner rolled margins of the basidiomes started to flatten. After removal from their substrates, these fruit bodies were weighed and yields were reported in grams per kg dry substrate. The ratio of grams (fresh) mushrooms harvested per kg dry substrate and expressed as a percentage gave the BE (Gupta et al., 2013b). Substrate dry matter loss was calculated from the difference between the initial and final dry weights of the substrate combinations.

### Chemical Analysis

The moisture content of substrates/substrate combinations/fresh mushroom fruit bodies was determined by heating them in an oven at 100-105^0^C until a constant weight was achieved. Incineration in the muﬄe furnace at 550 ± 5°C for 24 h gave the ash content (AOAC, 1995). Digital pH meter (Eutech Instruments pH 510) was employed to record the pH values.

All the substrate combinations/fruit bodies were freeze dried and subsequently powdered before carrying out the chemical analysis. CHN analyzer (CHNOS Elementar, Vario EL III) was used to estimate the carbon (C) and nitrogen (N) contents. Potassium (K) and phosphorus (P) were determined using flame photometer and spectrophotometer, respectively. Atomic absorption spectrophotometer (Perkin Elmer, Analyst 200) gave the elemental composition of iron and zinc. Nitrogen content when multiplied by a factor of 6.25 gave the crude protein content of WS and BGS. For mushroom fruit bodies, crude protein was calculated by multiplying their nitrogen content by a factor of 4.38 (Gothwal et al., 2012). Fat content was determined using Soxhlet apparatus and Anthrone method was used to determine the total soluble sugars (Thimmaiah, 2006). Total energy was calculated according to the following equation: Energy (KJ) = 17 × (g protein + g carbohydrate) +37 × (g lipid) (Beluhan and Ranogajec, 2011). Values reported are an average of three determinations.

### Sugar Composition

Sugar composition was estimated by extracting freeze dried mushroom powder with ethanol. About 300 mg of dry mushroom powder was shaken with 50 mL of 80% aqueous ethanol for 45 min at ambient temperature followed by filtering through Whatman No. 4 paper. To ensure complete extraction, the entire procedure was repeated approximately five times with 5 mL of aqueous ethanol each time. The ethanol extracts were pooled together and dried using a rotary-evaporator and the residue re-dissolved in deionized water to a final volume of 6 mL (Gupta et al., 2013b). The aqueous extracts were passed through 0.45 μm filter prior to injecting 20 μL of the sample into HPLC (Waters Corp., Milford, MA, USA), fitted with Waters e 2695 separation module, an auto injector (20 μL loop), ELSD detector (Waters 2424) and an amino column (Waters, 250 mm × 4.6 mm; 5 μm). The drift tube and nebulizer temperature were maintained at 80 and 48°C, respectively. Nitrogen, at a pressure of 25 psi, was used as the carrier gas. Mobile phase was acetonitrile:deionized water (85:15; v/v) at a flow rate of 0.75 mL min^-1^. Run time was 40 min. Results are expressed in g 100g^-1^dw. The sugars were identified by comparing the relative retention times of sample peaks with the known standards (Sigma chemicals, St. Louis, MO, USA).

### Amino Acid Composition

To analyze the amino acid composition, 1 g freeze dried mushroom powder was digested with 20 mL 6 N HCl at 120°C for 2 h, followed by dilution with 20 mL water and subsequent filtering through Whatman No. 4 paper. Derivatization of amino acid hydrolysates was done using phenylisothiocyanate (Gupta et al., 2013b). The derivatized samples were passed through 0.45 μm filter prior to injecting 20 μL of the sample into HPLC, equipped with a fluorescence detector (Waters, 2475; λ_ex_ = 285 nm; λ_em_ = 354 nm) and aAccQ Tag amino acid analysis column (Waters, 250 mm × 4.6 mm; 5 μm). Eluent A as acetate-phosphate buffer and eluent B as 60% acetonitrile:water were used as the mobile phases. During acid digestion of protein samples, asparagine, and glutamine were hydrolyzed to aspartic acid and glutamic acid, respectively, and hence their values are reported here.

### Analysis for Saponin Residues

Mushroom fruit bodies and spent were analyzed for the presence of any saponin residues. They were repeatedly extracted with ethanol followed by partitioning into the butanol phase. The butanol extracts were pooled, concentrated and compared with mahua saponins using HPLC equipped with a PDA detector (λ_max_ = 214 nm) and a RP-18 column (Waters, 4.6 mm × 250 mm; 5 μm). Methanol and deionized water in ratio of 65:35(v/v), at a flow rate of 0.5 mL min^-1^, was used as the mobile phase (Gupta et al., 2013b).

### Degradation of Complex Molecules in Spent

The spent samples were analyzed for the degradation of complex molecules, viz., celluloses, hemicelluloses, and lignin as a result of *Lentinus* colonization. *FTIR* spectra were recorded for dry and powdered spent samples on Perkin Elmer Spectrum One FTIR spectrometer and comparisons were made with un-inoculated WS as well as the inoculated WS control. Standard protocols were followed to estimate them quantitatively (Thimmaiah, 2006).

### Statistical Analysis

The experimental data has been collected in triplicates and the results expressed by their mean values and standard deviation (SD). One way analysis of variance (ANOVA) was done (SPSS; version 18.0) and significance of difference determined according to Duncan’s multiple range test (DMRT). *p*-values ≤ 0.05 were considered to be statistically significant. Principle component analysis (PCA) were performed using Microsoft^®^ Excel^®^ add-in Multibase package (Numerical Dynamics, Japan) to visualize data and the underlying factors that explain the pattern of correlations within a set of observed variables.

## Results

### Characteristics of Substrate

The composition of WS and BGS- T_1_, T_2_, T_3_, and T_4_ used in the present study has been mentioned in **Table 1**. It can be seen that BGS are a good source of mineral nutrients, especially nitrogen (1.48–2.75%). Moisture content for all substrate combinations varied between 59.48 and 60.62%. Data on pH shows that WS is slightly acidic in nature, and BGS alkaline in nature. On mixing, pH for the designed substrate combinations ranged between 6.87 and 7.04. This pH range has been found to be desirable for mushroom growth (Gothwal et al., 2012; Gupta et al., 2013b).

**Table 1:** Substrate analysis.

Characteristic	Wheat straw (WS)	T_1_	T_2_	T_3_	T_4_
Moisture (%)	61.45 (1.53)	6.8 (0.23)ˆ*	6.25 (0.27)ˆ*	6.18 (0.16)ˆ*	6.32 (0.34)ˆ*
N (%)	1.16 (0.02)	1.48 (0.02)	2.23 (0.04)	2.75 (0.12)	2.13 (0.05)
P (%)	0.35 (0.14)	0.23 (0.15)	0.26 (0.21)	0.37 (0.22)	0.30 (0.12)
K (%)	0.20 (0.13)	0.38 (0.12)	0.49 (0.15)	0.56 (0.14)	0.51 (0.13)
Fe (ppm)	315.10 (0.12)	75.23 (0.13)	446.50 (0.07)	465.45 (0.15)	525.37 (0.04)
Zn (ppm)	120.25 (0.15)	121.30 (0.05)	145.50 (0.12)	130.45 (0.05)	162.50 (0.03)
Celluloses (%)	34.23 (1.14)	18.15 (1.14)	14.35 (0.75)	12.85 (0.46)	12.65 (0.77)
Hemicelluloses (%)	26.18 (2.31)	20.86 (0.73)	14.20 (0.82)	13.58 (0.65)	10.82 (0.56)
Lignin (%)	17.5 (0.32)	7.42 (0.52)	5.75 (0.95)	5.62 (0.74)	4.75 (0.69)
Saponins (%)	0.0	0.0	2.92 (0.78)	1.32 (0.85)	2.26 (0.54)
C/N ratio	34.22	18.2	12.44	12.21	12.50
pH	6.68 (0.02)	7.46 (0.02)	7.38 (0.02)	7.32 (0.02)	7.35 (0.02)

*^∗^Values are indicated for dried biogas slurries. Numbers in brackets indicate standard deviation. T_*1*_ = Biogas slurry (BGS) of CD; T_*2*_ = BGS of 50 (CW DMC):50 (CD); T_*3*_ = BGS of 50 (HW DMC):50 (CD); T_*4*_ = BGS of 75 (HW DMC):25 (CD)*

**FIGURE 1:**
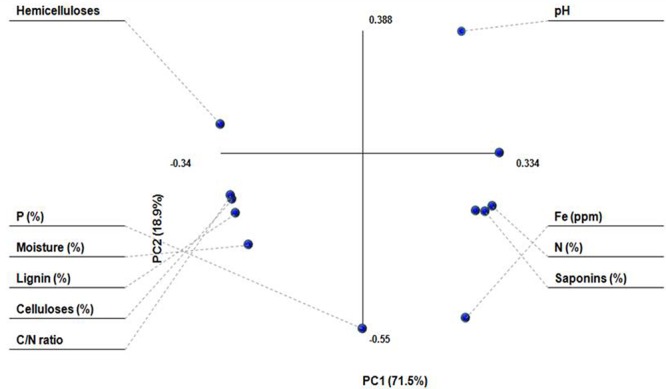
**Principle component analysis (PCA) of substrates**.

Principal component analysis was performed to see the pattern of correlation within set of observed variables and how these different variables varied in different treatments (**Figure 1**). PC1 and PC2 represented 90% of total variation in the observed variables. The dots for moisture (%), lignin (%), celluloses (%), and C/N ratio were very close to each other and were located on left hand side of the first component, showing negative correlation from other parameters. Fe, N, and saponins, on the other hand, were located on right hand side of the PC1 and showed positive correlation.

### Yield of Fruit Bodies, Substrate BE and Dry Matter Loss

Use of BGS as supplement to WS significantly enhanced the *Lentinus* fruit body yield as compared to WS control. A maximum yield of 1155 gkg^-1^ dry substrate was obtained from the treatment WS+20% T_3_, giving a BE of 115.5% and an increase of 95.10% over the control WS substrate. This was followed by the treatments WS+20% T_4_ (BE = 102.5%), WS+10% T_3_ (BE = 98.65%), WS+10% T_4_ (BE = 95.35%), WS+20% T_2_ (BE = 92.50%), WS+10% T_2_ (BE = 85.65%), and others in the order.

The formation of primordia on BGS supplemented WS substrates was observed from 21st to 24th day, with T_3_ supplemented substrates showing the earliest onset. The color of mushroom fruit bodies varied from white to light brown and was not affected by BGS supplementation. Loss in substrate dry matter ranged between 43.03 and 56.63%, with maximum for WS+20% T_3_ substrate combination, and minimum for the control.

### Nutrient Content of Fruit Bodies

Proximate analysis of mushroom fruit bodies, harvested from BGS supplemented substrates, revealed their moisture, ash and fat content in the range of 89.56-91.47, 7.63-8.04, and 1.81-2.01%, respectively (**Table 2**). Fat content of these fruit bodies was found to be significantly (*p* ≤ 0.05) lower than the control. **Table 2**, also summarizes the protein and total soluble sugar content of fruit bodies harvested from various combinations. Crude protein varied between 29.60 and 38.86%, showing a significant (*p* ≤ 0.05) increase on supplementation of WS (control) with BGS. Total soluble sugars in fruit bodies varied between 29.06 and 32.33%, with control showing the highest values. Based on proximate analysis, energy obtained from fruit bodies varied between 1122.30 and 1220.26 KJ. Overall, the data presented in the **Table 2**, showed the significant (*p* ≤ 0.05) increase in the value of protein, total soluble sugars, fat and energy in various combination of WS and BGS over control.

**Table 2:** Proximate composition of *Lentinus sajor-caju* fruit bodies harvested from WS supplemented with biogas slurries of cow dung and detoxified mahua cake.

Trt	Protein (%)				Total soluble sugars (%)				Fat (%)				Energy (kJ)			
WS	29.60^AaPi^ (0.36)				32.33^CcRiii^ (0.15)				2.01^CbQii^ (0.02)				1127.48^AaPi^ (4.86)			
**WS+**	**T1**	**T2**	**T3**	**T4**	**T1**	**T2**	**T3**	**T4**	**T1**	**T2**	**T3**	**T4**	**T1**	**T2**	**T3**	**T4**
**10%**	32.6^C^	35.6^c^	36.23^R^	36.60^iii^	30.93^A^	29.63^a^	29.06^p^	29.6^i^	1.86^B^	1.84^a^	1.84^p^	1.816^i^	1149.06^B^	1177.17^c^	1185^R^	1192.61^iii^
	(0.36)	(0.17)	(0.25)	(0.36)	(0.07)	(0.15)	(0.11)	(0.17)	(0.005)	(0.005)	(0.011)	(0.011)	(6.78)	(5.67)	(4.89)	(4.37)
**20%**	33.43^D^	36.93^d^	38.86^s^	37.24^1V^	31.06^A^	29.4^a^	29.26^PQ^	29.7^i^	1.826^A^	1.83^a^	1.813^p^	1.826^i^	1164.08^C^	1195.37^d^	1220.26^S^ 1205.62^1v^	
	(0.40)	(0.11)	(0.25)	(0.25)	(0.11)	(0.17)	(0.25)	(0.17)	(0.005)	(0.01)	(0.005) (0.005)		(7.62)	(7.92)	(2.43)	(2.33)
**30%**	30.5^B^	33.16^b^	34.61^Q^	33.76^ii^	31.43^B^	30.3^b^	29.46^Q^	30.1^ii^	1.87^B^	1.81^a^	1.826^P^	1.83^i^	1122.30^A^	1146.15^b^	1153.60^Q^	1153.32^ii^
	(0.50)	(0.28)	(0.53)	(0.25)	(0.11)	(0.45)	(0.05)	(0.17)	(0.005)	(0.005)	(0.011)	(0.005)	(8.77)	(8.59)	(7.20)	(2.07)

*Means followed by a different superscript in each column are statistically different from each other (*p* ≤ 0.05) using DMRT. For WS, four subscripts are for T_*1*_, T_*2*,_ T_*3*_, and T_*4*,_ respectively. Numbers in brackets indicate standard deviation. T_*1*_= BGS of CD; T_*2*_ = BGS of 50 (CW DMC):50 (CD); T_*3*_ = BGS of 50 (HW DMC):50 (CD); T_*4*_ = BGS of 75 (HW DMC):25 (CD).*

### Sugar Composition

From the above results, it was seen that supplementation of WS with 20% BGS was optimum for attaining maximum yield. Therefore, substrate combinations containing WS+20% each of T_1_, T_2_, T_3_, and T_4_, were selected and soluble sugar contents of fruit bodies harvested from them were evaluated. Data presented in **Table 3**, showed significant (*p* ≤ 0.05) reduction of total content of soluble sugars in fruit bodies of WS supplemented with 20% T1 (4.82), 20% T2 (4.97), 20% T3 (4.09), and 20% T4 (4.27) from 7.54 g 100g^-1^dw (in WS control).

**Table 3:** Soluble sugar composition (g 100g^-1^dw) of *L. sajor-caju* fruit bodies harvested from WS supplemented with biogas slurries of cow dung (CW) and detoxified mahua cake.

Sugar (g 100g^-1^dw)	WS (control)	WS supplemented with			
		20% T_1_	20% T_2_	20% T_3_	20% T_4_
Glucose	1.58^a^ (0.07)	2.26^c^ (0.06)	2.34^d^ (0.05)	2.14^b^ (0.08)	2.31^d^ (0.02)
Mannitol	0.10^a^ (0.02)	0.46^d^ (0.02)	0.31^c^ (0.03)	0.08^a^ (0.04)	0.21^b^ (0.04)
Trehalose	5.86^e^ (0.07)	2.10^c^ (0.04)	2.31^d^ (0.04)	1.87^ab^ (0.03)	1.74^a^ (0.05)
**Total**	**7.54^e^ (0.06)**	**4.82^c^ (0.05)**	**4.97^d^ (0.03)**	**4.09^a^ (0.04)**	**4.27^b^ (0.03)**

*Means followed by a different superscript in each row are significantly different from each other (p ≤ 0.05) using DMRT. Numbers in brackets indicate standard deviation. T_1_ = BGS of CD; T_2_ = BGS of 50 (CW DMC):50 (CD); T_3_ = BGS of 50 (HW DMC):50 (CD); T_4_ = BGS of 75 (HW DMC):25 (CD).*

### Amino Acid Composition

*Lentinus* fruit bodies harvested from above selected substrate combinations were also evaluated for their amino acid content (**Table 4**). A total of sixteen amino acids (including essential ones) were detected by HPLC analysis. Data presented in the **Table 4** showed a significant (*p* ≤ 0.05) increase in amino acids content (g 100g^-1^dw) in case of WS supplemented with 20% BGS of CW and detoxified mahua cake (T_1_, T_2_, T_3_, T_4_) in comparison to the WS (control). Supplementary Figure S1 shows a representative HPLC chromatogram of amino acids, present in fruit bodies of substrate combination WS+20% T_3_.

**Table 4:** Amino acid composition (g 100g^-1^dw) of *L. sajor-caju* fruit bodies harvested from WS supplemented with biogas slurries of cow dung and detoxified mahua cake.

Amino acid (g 100g^-1^dw)	WS (control)	WS supplemented with			
		20% T_1_	20% T_2_	20% T_3_	20% T_4_
Aspartic acid	2.20^a^ (0.11)	3.60^d^ (0.05)	4.14^e^ (0.05)	2.91^b^ (0.07)	3.24^c^ (0.03)
Serine	1.51^b^ (0.05)	1.12^a^ (0.03)	1.87^c^ (0.04)	2.63^e^ (0.03)	1.97^d^ (0.02)
Glutamic acid	2.47^a^ (0.02)	4.50^c^ (0.03)	5.50^d^ (0.03)	6.81^e^ (0.05)	3.82^b^ (0.04)
Glycine	2.02^b^ (0.03)	0.93^a^ (0.02)	2.66^d^ (0.06)	2.23^c^ (0.06)	3.37^e^ (0.01)
Histidine^∗^	1.02^b^ (0.05)	2.39^d^ (0.03)	0.68^a^ (0.05)	1.03^b^ (0.04)	1.18^c^ (0.05)
Arginine+ Threonine^∗^	1.64^a^ (0.10)	2.83^c^ (0.16)	2.32^b^ (0.03)	3.56^d^ (0.07)	2.76^c^ (0.05)
Alanine	3.35^a^ (0.17)	3.85^b^ (0.15)	5.03^d^ (0.04)	3.30^a^ (0.04)	4.49^c^ (0.09)
Proline	2.94^c^ (0.03)	2.61^b^ (0.01)	3.34^d^ (0.05)	1.76^a^ (0.05)	1.72^a^ (0.02)
Cysteine	0.39^c^ (0.02)	0.18^a^ (0.03)	0.38^c^ (0.01)	0.28^b^ (0.01)	0.39^c^ (0.03)
Tyrosine	1.53^b^ (0.04)	1.95^c^ (0.05)	1.07^a^ (0.05)	2.31^d^ (0.06)	2.32^d^ (0.01)
Valine^∗^	2.51^d^ (0.02)	0.99^a^ (0.02)	2.18^b^ (0.03)	2.29^c^ (0.01)	2.30^c^ (0.07)
Methionine^∗^	0.82^b^ (0.01)	0.61^a^ (0.03)	Not detected	0.95^c^ (0.02)	0.93^c^ (0.01)
Lysine^∗^	1.57^b^ (0.02)	0.13^a^ (0.02)	1.98^d^ (0.03)	1.83^c^ (0.03)	2.18^e^ (0.01)
Isoleucine^∗^	1.28^b^ (0.01)	1.08^a^ (0.02)	1.03^a^ (0.01)	1.35^b^ (0.01)	1.28^b^ (0.02)
Leucine^∗^	2.04^b^ (0.02)	1.05^a^ (0.01)	2.27^c^ (0.05)	2.82^e^ (0.05)	2.41^d^ (0.04)
Phenylalanine^∗^	2.31^b^ (0.03)	2.37^b^ (0.05)	1.57^a^ (0.01)	2.72^d^ (0.03)	2.55^c^ (0.02)
**Total**	**29.60^a^ (0.17)**	**30.20^b^ (0.25)**	**36.60^c^ (0.13)**	**38.86^c^ (0.15)**	**36.93^b^ (0.18)**

*^∗^Essential amino acids. Means followed by a different superscript in each row are significantly different from each other (*p* ≤ 0.05) using DMRT. Numbers in brackets indicate SD. T_1_ = BGS of CD; T_2_ = BGS of 50 (CW DMC):50 (CD); T_3_ = BGS of 50 (HW DMC):50 (CD); T_4_ = BGS of 75 (HW DMC):25 (CD).*

**FIGURE 2:**
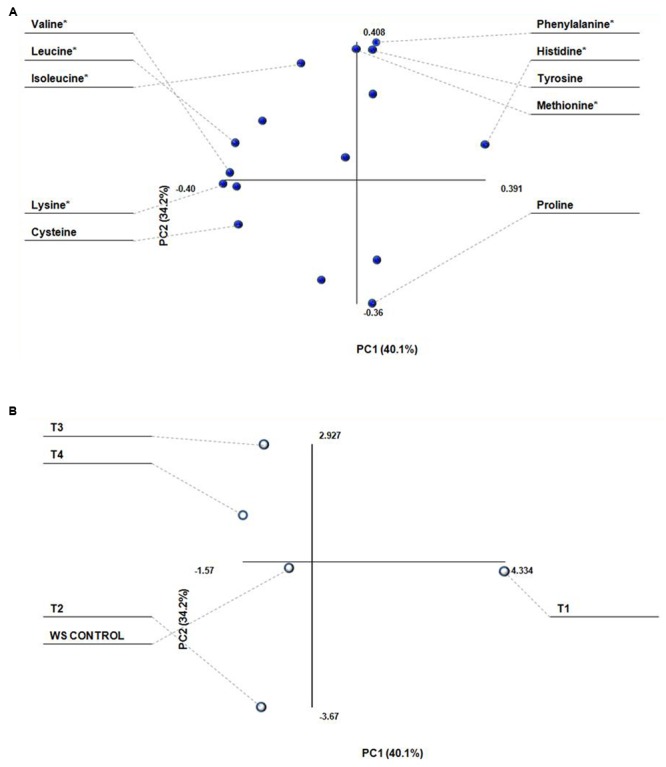
**Principle component analysis of amino acid composition (g 100g^-1^dw) of *Lentinus sajor-caju* fruit bodies harvested from wheat straw (WS) supplemented with biogas slurries of cow dung (CW) and detoxified mahua cake. (A)** Loading plot showing different amino acids as variables and, **(B)** Score plot showing different treatments as T1 = Biogas slurry (BGS) of CD; T2 = BGS of 50 (CW DMC):50 (CD); T3 = BGS of 50 (HW DMC):50 (CD); T4 = BGS of 75 (HW DMC):25 (CD). ^∗^Essential amino acid.

Amino acid data set was composed of sixteen measurements for each data point. In raw form, these data are difficult for analysis visually, but when reduced to two or three principal components, the visualization of the entire data set becomes traceable (**Figures 2A,B**). PCA generates two types of plot, score plot, and loading plot. The score plot shows the similarity or dissimilarity of samples (Treatments), and the loading plot represents summary of the variables (different amino acids). The two plots are complementary and can be superimposed. PCA of amino acids showed that, PC1 and PC2 represent 74% of total variation in the data set. Most of the amino acids (Lysine, histidine, valine, leucine cysteine, glycine, and serine) are negatively correlated, while phenylalanine, methionine, tyrosine, isoleucine, arginine, threonine, and serine are positively correlated. Among different treatments, T1 is most positively and WS is most negatively correlated with the amino acid contents.

### Mineral Content

The mineral contents of *Lentinus* fruit bodies harvested from different substrate combinations has been listed in **Table 5**. Nitrogen was present in highest amount with its content varying from 67.57 and 88.72 mgg^-1^dw. Potassium (K), phosphorous (P), iron (Fe), and zinc (Zn) were obtained in the ranges of 23.06-29.86 mgg^-1^dw, 11.23-15.01 mgg^-1^dw, 105.76-247.66 ppm, and 75.76-114.32 ppm, respectively. Overall, different mineral content of *L. sajor-caju* fruit bodies harvested from WS supplemented with biogas slurries of CW and detoxified mahua cake were significantly (*p* ≤ 0.05) higher over the WS (control).

**Table 5:** Mineral content of *L. sajor-caju* fruit bodies harvested from WS supplemented with biogas slurries of cow dung and detoxified mahua cake.

Trt	Nitrogen (mgg^-1^)				Phosphorous (mgg^-1^)				Potassium (mgg^-1^)				Iron (ppm)				Zinc (ppm)			
WS	67.57^AaPi^ (0.37)				11.23^AaPi^ (0.17)				23.26^BaPi^ (0.15)				105.76^AaPi^ (0.25)				75.76^AaPi^ (0.28)			
**WS+**	**T1**	**T2**	**T3**	**T4**	**T1**	**T2**	**T3**	**T4**	**T1**	**T2**	**T3**	**T4**	**T1**	**T2**	**T3**	**T4**	**T1**	**T2**	**T3**	**T4**
**10%**	74.42^C^ (0.57)	81.27^c^ (0.27)	32.71^R^ (0.40)	83.56^iii^ (0.57)	11.56^C^ (0.05)	13.36^c^ (0.11)	13.46^R^ (0.05)	12.38^iii^ (0.10)	23.46^C^ (0.05)	27.63^c^ (0.15)	28.72^R^ (0.17)	26.46^iii^ (0.057)	129.66^B^ (0.52)	185.93^b^ (0.32)	197.33^Q^ (2.08)	212.30^ii^ (0.15)	81.66^B^ (0.41)	88.77^b^ (0.35)	91.76^Q^ (2.08)	94.83ii (0.21)
**20%**	76.32^D^ (0.64)	84.31^d^ (0.18)	88.72^S^ (0.40)	85.02^iv^ (0.40)	11.76^D^ (0.05)	14.26^d^ (0.11)	15.01^S^ (0.10)	13.36^iv^ (0.05)	24.63^D^ (0.15)	29.44^d^ (0.15)	29.86^S^ (0.11)	27.16^iv^ (0.15)	132.83^C^ (0.45)	189.66^c^ (0.52)	200.66^R^ (0.18)	247.66^iv^ (0.31)	82.76^C^ (0.25)	89.96^c^ (0.36)	98.57^R^ (1.08)	114.32^iv^ (0.51)
**30%**	69.61^B^ (0.80)	75.70^b^ (0.46)	79.01^Q^ (0.86)	77.07^ii^ (0.40)	11.35^B^ (0.05)	11.86^b^ (0.15)	11.96^Q^ (0.05)	11.90i^i^ (0.10)	23.06^A^ (0.05)	24.66^b^ (0.15)	25.91^Q^ (0.11)	25.43^ii^ (0.1)	134.06^C^ (0.22)	187.83^c^ (0.46)	206.66^S^ (0.32)	223.63^iii^ (0.15)	86.24^C^ (0.76)	93.57^c^ (0.50)	102.66^S^ (0.33)	91.63^iii^ (0.64)

*Means followed by a different superscript in each column are statistically different from each other (*p* ≤ 0.05) using DMRT. For WS, four subscripts are for T_*1*_, T_*2*,_ T_*3*_, and T_*4*,_ respectively. Numbers in brackets show standard deviation. T_*1*_ = BGS of CD; T_*2*_ = BGS of 50 (CW DMC):50 (CD); T_*3*_ = BGS of 50 (HW DMC):50 (CD); T_*4*_ = BGS of 75 (HW DMC):25 (CD).*

### Analysis for Saponin Residues- HPLC Studies

**FIGURE 3:**
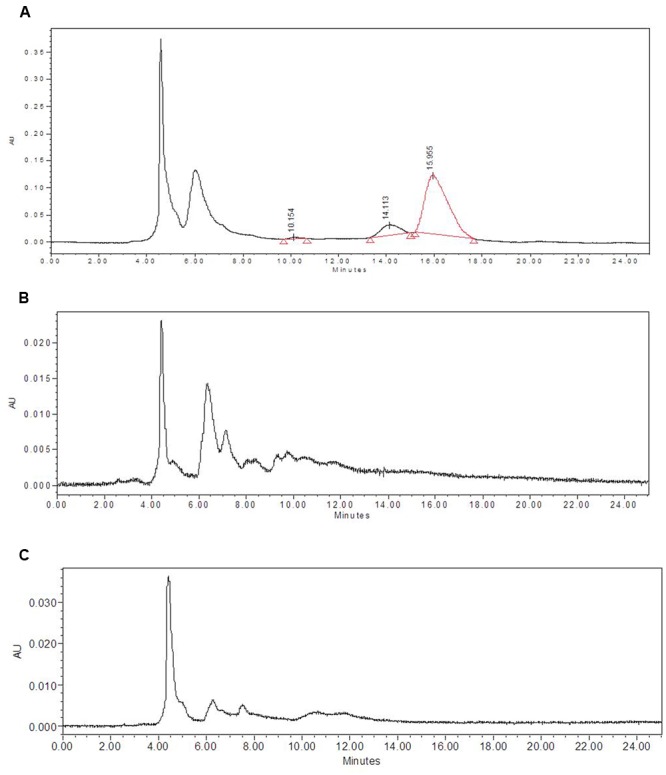
**Analysis of saponin residues in mushroom fruit bodies **(A)** HPLC, chromatogram of butanol extract (saponins) of mahua cake.** HPLC chromatogram of butanol extract of *L. sajor-caju* fruit bodies harvested from **(B)** WS and **(C)** WS+20% T_2_.

Biogas slurries of DMC and CD used in the present study contained small amounts of residual saponins (**Table 1**). Since mahua saponins are known to possess some toxicity, it was important to check their presence in mushroom fruit bodies and spent. The latter were extracted with ethanol followed by subsequent partitioning into the butanol phase. The pooled, concentrated butanol fractions were compared with mahua saponins. HPLC chromatogram of mahua saponins showed three peaks with characteristic retention times 10.154, 14.113 and 15.995 min (**Figures 3A–C**). However, no peaks were detected at (or around) above-mentioned retention times in chromatograms of butanol extract of fruit bodies, harvested from WS and WS+20% T_2_ substrates. The latter substrate combination was chosen as it contained maximum saponin content, amongst the treatments designed (**Table 1**); higher concentrations were not considered because of decrease in yield/BE. Similar observations were recorded from other fruit bodies and spent.

### Degradation of Complex Molecules and Manurial Value of Spent

Over a span of 50 days, complex molecules-celluloses, hemicelluloses and lignin were degraded to varying extents by *Lentinus*, in different substrate combinations. These changes were monitored via *FTIR.* Supplementary Figure S2 shows a representative *FTIR* spectrum of un-inoculated WS, WS+20% CD and WS+20% T_3_ spent. Quantitatively, a decrease of 22.20-32.40% in celluloses, 14.13-23.05% in hemicelluloses, and 27.43-39.23% in lignin was observed.

## Discussion

Supplementation of WS with BGS of DMC significantly (*p* ≤ 0.05) enhanced the *Lentinus* fruit body yield as compared to WS control. It was observed that *Lentinus* fruit body yield increased with the addition of 10 and 20% BGS to WS and then decreased on further addition of 30% BGS. This may be due to enhanced supply of nutrients available from slurry(s) to the fungus which might have prompted its growth. Thereafter, excess form of these nutrients and/or degradation products might have become toxic to *Lentinus* fungus (Gothwal et al., 2012; Gupta et al., 2013b). Another possible reason could be excessive moisture accumulation in the mushroom beds, which might have restricted mycelium growth and eventually sporophore development, in spite of the availability of sufficient minerals from BGS (Namasivayam and Yamuna, 1995). In all the cases, the order of performance varied as: T_3_ > T_4_ > T_2_ > T_1_. This order followed the same pattern as observed for biogas production from their respective combinations. It is already known that during biomethanation process, complex organic matter is broken down into simpler forms (Gupta et al., 2013a). Greater production of biogas would imply greater availability of nutrients which might be the reason for the observed trend.

Maximum yield of 1155 gkg^-1^ dry substrate, obtained from treatment WS+20% in our study, is greater than reported yield of 482 gkg^-1^ *V. volvacea* obtained from rice straw supplemented with CD biomanure (1:1 ratio) and 485 gkg^-1^, *P. sajor-caju* from hot water sterilized rice straw supplemented with jute caddis biomanure (1:2 ratio) (Banik and Nandi, 2000, 2004). Maximum BE (115.5%) obtained in the present study is also greater than reported values of 49.7, 99.1, 89.5, and 98.4% for *P. sajor-caju* mushrooms cultivated on pre-sterilized (2% formalin + 0.1% bavistin in cold water) rice straw and rice straw supplemented with CD, jute caddis, and poultry litter biomanures, respectively (Banik and Nandi, 2004). However, yield of 2.488 kgkg^-1^ in *P. flabellatus* has been reported from paddy straw supplemented with residual spent from biogas plants, containing leaf biomass (Gangulli and Chanakya, 1994), and 2.4 kgkg^-1^ *P. ostreatus* has been recorded from paddy straw supplemented with digester liquid from a biomass biogas plant (Ashwath et al., 2016). The addition of spent biogas provides a good source of nutrients, especially nitrogen and phosphorous, which boosts the mushroom productivity (Gangulli and Chanakya, 1994; Banik and Nandi, 2000, 2004; Ashwath et al., 2016).

Besides differences in nutrient content of BGS used in the present study, an important difference was in their saponin content (**Table 1**). Toxicity of mahua saponins towards different strains of fungi, bacteria, and other pathogens has already been reported (Lalitha and Venkataraman, 1991; Saha et al., 2010; Gothwal et al., 2012). It was envisaged that their presence was inhibitory to *Lentinus* as well. Supplementary Figure S3 shows the growth inhibition of *Lentinus* by 0.1, 0.2, and 0.5% mahua saponins studied by food poisoned technique (Saha et al., 2010).

Results on primordia formation of *Lentinus* fruit bodies on supplemented WS substrates could be corroborated by literature, where different *Lentinus* spp. showed primordial formation from 21th to 30th days (Ragunathan et al., 1996; Ragunathan and Swaminathan, 2003). Ashwath et al. (2016) observed that time taken for *P. ostreatus* pin head formation on paddy straw was greatly reduced with the addition of biogas digester liquid to the latter. After colonization by *Lentinus*, a significant reduction in dry matter of the substrates was observed. The dry matter got partly assimilated into mushroom fruit bodies and partly lost to atmosphere as carbon dioxide due to mushroom respiration (Gupta et al., 2013b; Ashwath et al., 2016). This gave a considerable mass reduction, along with an efficient method for waste disposal and utilization.

Proximate analysis of mushroom fruit bodies showed that they are rich in protein and soluble sugars (Bisaria et al., 1987; Ragunathan et al., 1996; Gothwal et al., 2012; Gupta et al., 2013b). Mushrooms fruit bodies harvested from WS+T_3_ substrate combinations had higher protein as compared to other treatments (**Table 2**). This might be attributed to higher nitrogen content of T_3,_ as studies have shown that protein content of mushrooms varies with differential nutrient supply of the chosen substrate (Gothwal et al., 2012; Naraian et al., 2016). Paul et al. (2015) showed that nutrient composition of *P. florida* varied significantly with the nature of five different saw dust substrates chosen. The maximum value of protein content, 38.86% recorded from WS+20% T3 substrate combination, is higher than the reported literature values of 29.81, 33.57, and 33.84% protein in *P. sajor-caju* fruit bodies harvested from rice straw supplemented with CD, poultry litter and jute caddis biomanures, respectively (Banik and Nandi, 2004). The decrease in the protein content at higher concentrations (30%) of BGS supplements might be due to inhibition of fungal growth as mentioned above and hence the inability of *Lentinus* fungus to efficiently degrade the substrate and utilize its nutrients (Gupta et al., 2013b). Supplementation of WS with different levels of BGS did not increase the sugar content compared to control (**Table 2**). Banik and Nandi (2004) also reported a significant increase in protein content, while a reverse trend for carbohydrates in *V. volvacea* and *P. sajor-caju* mushrooms, when cultivated on rice straw supplemented with different biomanures. This indicated the reorientation of metabolic pathways toward protein synthesis on supplementation (Banik and Nandi, 2000). Based on proximate analysis, the values of energy obtained are fairly large than those reported in the literature (Ragunathan and Swaminathan, 2003; Gupta et al., 2013b).

Total content of soluble sugars in fruit bodies varied from 4.09 to 7.54 g 100g^-1^dw (**Table 3**). Trehalose (1.74–5.86 g 100g^-1^dw) and glucose (1.58–2.34 g 100g^-1^dw) were present in highest amounts and mannitol in least amount (0.08–0.46 g 100g^-1^dw). It was interesting to see that even though, the individual sugar content varied; the treatments had significantly lower sugar content compared to control. Beluhan and Ranogajec (2011) reported values of 15.01, 9.82, and 1.79 g 100g^-1^ for glucose, mannitol and trehalose, respectively, for *P. ostreatus*. Yang et al. (2001) reported values of 11.6 and 10.6 mgg^-1^dw for glucose, 24.6 and 3.6 mgg^-1^dw for mannitol and 28.6 and 2.7 mgg^-1^dw for trehalose for *P. cystidiosus* and *P. ostreatus*, respectively.

The total content of sixteen amino acids in fruit bodies ranged between 29.60 and 38.86 g 100g^-1^dw, with alanine and glutamic acid present in highest amounts (**Table 4**). Cysteine and methionine, on the other hand, were present in least amounts. All fruit bodies possessed essential amino acids (leucine, histidine, isoleucine, valine, phenylalanine, lysine, etc.) in fair good amounts. A number of authors have reported mushrooms to be an excellent source of amino acids. A total amino acid content of 66.67 g 100g^-1^, with glutamic acid in highest amounts, has been reported in *P. sajor-caju* fruit bodies harvested from WS (Bisaria et al., 1987). Beluhan and Ranogajec (2011) observed free amino acid content between 0.01 and 41.09 mgg^-1^dw for Croatian wild variety of *P. ostreatus* and Yang et al. (2001) reported values between 0.02 and 3.94 mgg^-1^dw for the two *Pleurotus* species studied.

Minerals such as nitrogen (N), Potassium (K), Phosphorous (P), Iron (Fe), and Zinc (Zn) were also present in mushroom fruit bodies (**Table 5**). It was observed that nitrogen content of fruit bodies increased significantly (p ≤ 0.05) with increasing concentrations of BGS (except at higher concentrations). Fruit bodies harvested from WS supplemented with T_3_ substrates were richer in nitrogen than those harvested from WS supplemented with other BGS. This might be attributed to higher nitrogen content of T_3_substrate (**Table 1**). Biogas slurries have been known to be effective in enhancing the mineral contents of mushrooms. Banik and Nandi (2000, 2004) reported values of 1.18–1.27% and 0.25–1.05% phosphorous (P), 4.80–5.68% and 2.27–4.35% potassium (K), 230–301 ppm and 25–59.9 ppm iron (Fe) and 94–123 ppm and 12–94.2 ppm zinc (Zn) in *V. volvacea* and *P. sajor-caju* mushrooms, respectively, harvested from biomanure supplemented rice straw.

HPLC studies confirmed the absence of saponin residues in fruit bodies and spent and suggested that, even though, their presence was inhibitory to *Lentinus* fungus; latter was able to degrade them effectively (Gupta et al., 2013b). Lalitha and Venkataraman (1991) also reported initial growth inhibition of *Trichoderma viride* by *Madhuca butraceae* saponins, followed by growth resumption at a later period, suggesting the development of fungal resistance by saponin degradation. Enzymatic action of *L. sajor-caju* might have been responsible for the degradation of mahua saponins. Such observations can be corroborated by literature as well. Ruiz-Rodriguez et al. (2010) suggested the role of phenol oxidases in destruction of olive related phenolics in oyster mushrooms, when cultivated on WS supplemented with olive waste. Shashirekha et al. (2002) also observed the absence of gossypol polyphenolics in *P. sajor-caju* fruit bodies, when cultivated on cotton seed cake supplemented rice straw.

*FTIR* analysis (Supplementary Figure S2) revealed the changes in relative intensities of spectral bands associated with complex molecules- celluloses, hemicelluloses and lignin, in different substrate combinations. Absorption at 3450 cm*^-^*^1^ corresponded to O–H stretching (hydrogen bonded) and at 2920 cm*^-^*^1^ to C–H stretching. Fingerprint region between 1800 and 600 cm*^-^*^1^showed the presence of many well-defined peaks: 1732 cm^-1^ for unconjugated C = O in xylans (hemicelluloses), 1507 cm*^-^*^1^ for aromatic skeletal in lignin, 1426 cm^-1^ for C–H deformation in lignin and carbohydrates, 1370 cm*^-^*^1^ for C–H deformation in celluloses and hemicelluloses, 1320 cm*^-^*^1^ for C–H vibration in celluloses and C_l_–O vibration in syringyl derivatives, 1158 cm*^-^*^1^ for C–O–C vibration in celluloses and hemicelluloses and 898 cm*^-^*^1^ for C–H deformation in celluloses (Gupta et al., 2013b). On comparing the *FTIR* spectra of WS+20% CD and WS+20% T_3_ spent with un-inoculated WS, a substantial decrease in the intensity of abovementioned peaks was observed. This showed that the fungus (*Lentinus*) degraded celluloses, hemicelluloses and lignin in the substrates. This might be attributed to increased activity of cellulases, hemicellulases, and laccase, which are involved in their degradation (Shashirekha et al., 2002). It should be noted that during biomethanation of MC and CD, celluloses and hemicelluloses were degraded to substantial amounts and lignin was the least degraded component (Gupta et al., 2013b). Colonization by white rot fungus, *Lentinus*, led to a significant decrease in lignin content of the substrates which is beneficial from the view point of straw digestibility, hence improving its quality as cattle feed. Gangulli and Chanakya (1994) also reported a significant reduction in lignin content (>50%) of paddy straw substrates supplemented with residual spent from biogas plants.

After degradation of complex molecules, an increase in the nutrient content of mushroom spent (21.46-26.61% increase in nitrogen, 10.84-14.32% in phosphorous and 12.58-21.26% in potassium) was obtained. Gangulli and Chanakya (1994) and Ashwath et al. (2016) also reported an increase in nitrogen/protein content of paddy straw substrates, when supplemented with residual spent from biogas plants, as a result of *L. florida* cultivation. The spent was therefore found to be rich in simpler nutrients along with absence of any saponin residues. This is beneficial from view point of using it as a quality manure or cattle feed with improved dry matter digestibility (Shashirekha et al., 2002).

## Conclusion

The present study is a pioneer attempt that demonstrates the use of BGS of DMC and CD to improve the yield and nutritional value of *Lentinus* on WS. Supplementation with 20% BGS was found to be optimum for obtaining maximum yield, showing an increase of 95.1% over control. Fruit bodies were rich in proteins, soluble sugars, amino acids, minerals and were free from any saponin residues. Overall, the study proved beneficial for effective management of BGS along with producing edible and nutritive mushrooms, and spent with improved digestibility and feed quality.

## Author Contributions

AG performed the experiments and prepared the manuscript. SS designed the experiments and reviewed the manuscript. AK performed the analysis of data and reviewed the manuscript. PrA and PaA critically edited and formatted the manuscript.

## Conflict of Interest Statement

The authors declare that the research was conducted in the absence of any commercial or financial relationships that could be construed as a potential conflict of interest.
