# Supplementary figures and images for: Enhancing Nutritional Contents of Lentinus sajor-caju Using Residual Biogas Slurry Waste of Detoxified Mahua Cake Mixed with Wheat Straw
Source: Front Microbiol. 2016 Oct 13;7:1529. doi: 10.3389/fmicb.2016.01529 (PMC5062869; doi:10.3389/fmicb.2016.01529)

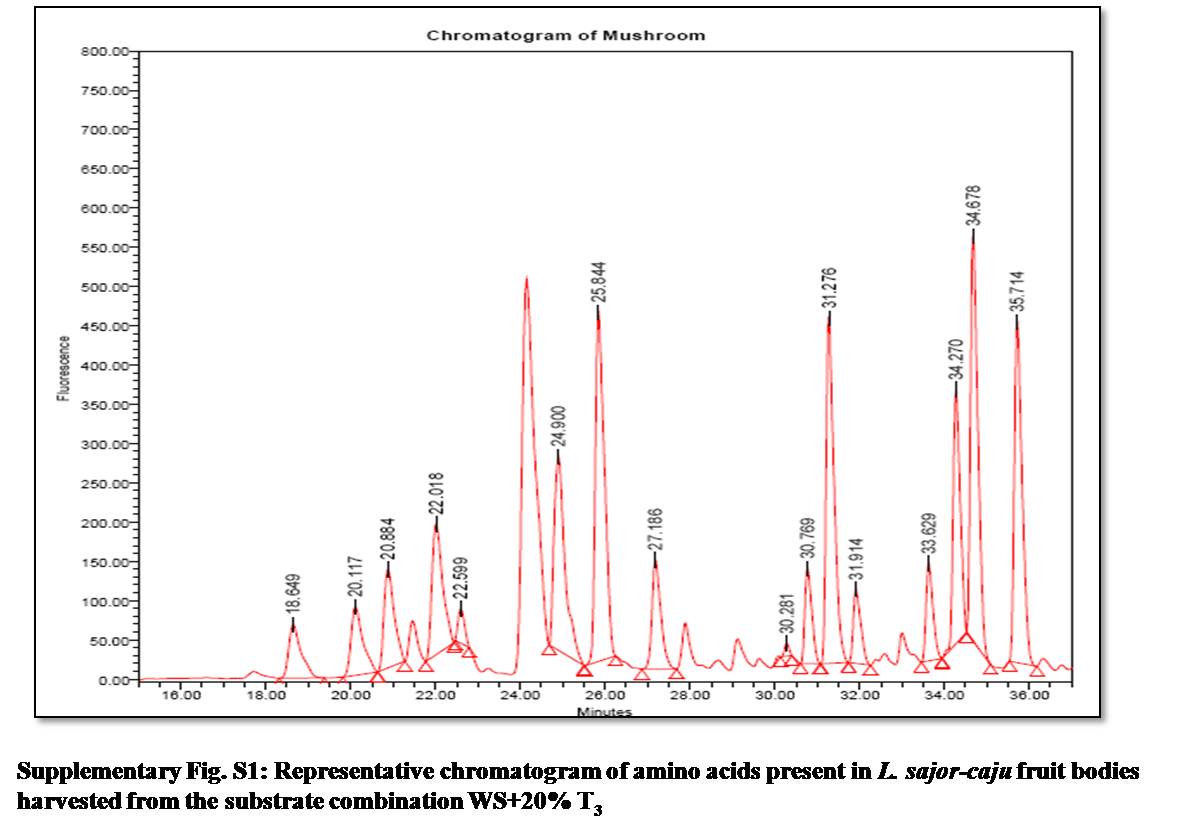

Supplement: Supplementary file 1 [file Image_1.JPEG]

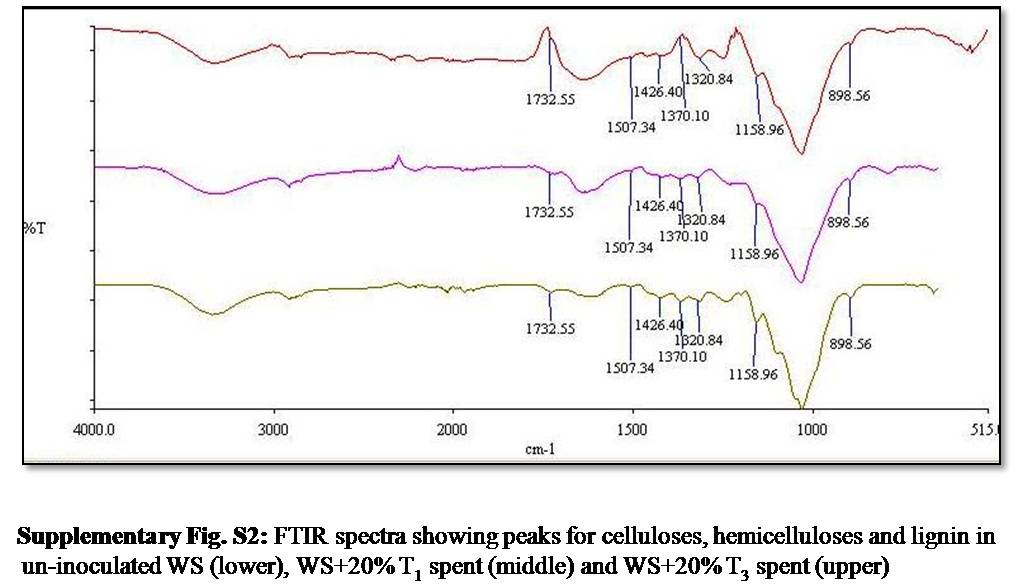

Supplement: Supplementary file 2 [file Image_2.JPEG]

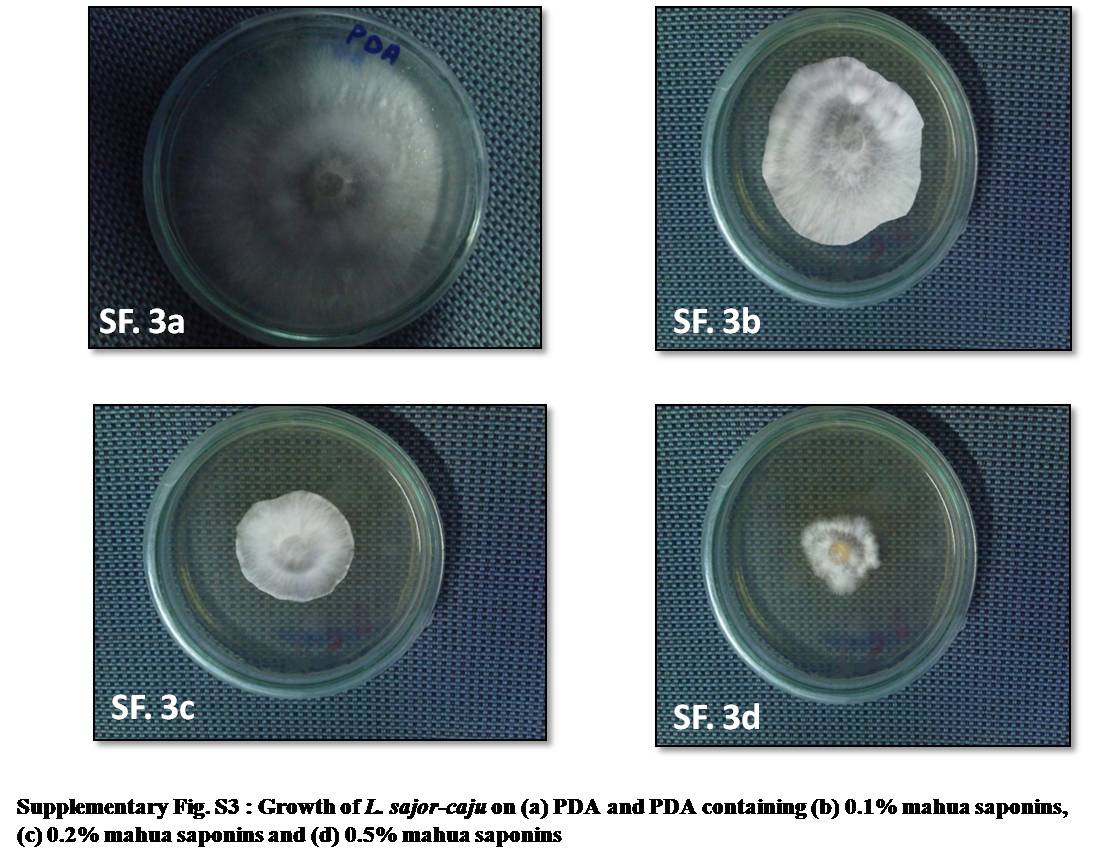

Supplement: Supplementary file 3 [file Image_3.JPEG]
